# Provings of Lachesis

**Published:** 1882-07

**Authors:** B. Fincke

**Affiliations:** Brooklyn, N. Y.


### PROVINGS OF LACHESIS.

B. Fincke, M. D., Brooklyn, N. Y.

1864, April 12th. B. F., 43 years old, took Lachesis 71 m (F.) 6 globules on the tongue. After two hours: on picking his teeth, spasm of the jaws so that he could close his mouth only with difficulty with spasmodic pains especially about the mandibular joints. Painful, fine drawing through the left side of the head from before backwards, and later some straining in the left ear.

1867. Mrs. F. took Lachesis 90 m (F.) and got an abominable taste with sensation as if mouth and pharynx were smeared over with it.

The following provings were obtained by holding the vials with the medicine in the hollow hand as long as any symptoms appeared. I was led to it by investigations concerning mesmerism, and did not at the time know of the beautiful experiment of Dr. O. Buchmann with life quicksilver, published in the *Homœopathic Vierteljahrschrift*, vol. 15, in 1864. You may imagine how pleased I was to have been guided to the same track as this celebrated homœopathician. I, also, at that time felt a great satisfaction at the experience, that the efficaciousness of even the highest potencies can be demonstrated at once with very sensitive subjects, such as was my good fortune to find. It is even so: "seek, and ye will find; knock, and it will be opened unto you." But, unfortunately, we knock at the head of the majority of the profession ever so long and it will not be opened unto us.

1868, June 22d. Mr. G., a professional magnetizer, tall and stout, took Lachesis M (F.)—M (F.) means always million; I cannot change my notation to please Drs. Skinner and Swan—in the hollow of his hand. He felt an itching on the skin of the hand which held the vial, like from the itch. It is to him as if the blood were running back from the hand to the arm with itching pain, as if it were in the blood. Confusion in the head.

After changing the vial to Lachesis 2 M (F.) the itching becomes milder, and extends as far as under the axilla. L. arm like lame interiorly.

*Dr. E. T. Richardson* took Lachesis 77 m (F.) in his right hand, and felt a warmth going up the right arm.

*Miss S.*, about 40 years old, tall, took Lachesis 2 M (F.) in her left hand, and noticed nothing. After five minutes, she took the vial in the right hand. Immediately sensation, as if one expects somebody with great joy. Glimmering before the eyes. Head heavy like lead, as if it were off around below, and would fall down. Lame in both arms. Inclination to vomit. Twitching at both superciliary ridges and malar bones, with burning. Head weak, cannot think any more, it takes away the understanding.

Now, she takes the vial in her left hand, and observes a singular motion in the upper part of the body, throbbing somewhere in the chest, but she cannot say where. Hacking cough. Eyes heavy. Heaviness of the occiput, drawing down, as if something heavy hung on to it. Twitching in the chest. Heat in face and ears.

Taking the vial in the hollow of both hands. Burning in her eyes. Pulse weak, slow. Tearing up the left forehead. Head weak, cannot think well. Sleepy, it closes the eyes. Now, she says, it changes. Grasping in both upper arms. Then, she says, it is done. This had lasted a quarter of an hour.

Took Lachesis M (F.) in the right hand. Throbbing in the chest with inclination to vomit and weakness of the head. Drawing down heaviness in the occiput. Hot ears. Sleepy. Heat in the head. The head gets still hotter. Burning in the head, especially around the forehead. Pulse full and slow. The heart-stroke intermits, the pulse gives a start, and then is imperceptible. Face yellow. Heaviness in the whole body. She must put away the vial, or else she falls asleep.

After now taking Opium Cm (F)., in both hands, everything is like blown away, the heaviness disappears, the heat likewise, as if it were wiped off from the forehead. After five minutes, she is as well as before, and feels quite cool and pleasant. But Opium exerts no further action.

July 31st. Miss S. held the newly-prepared Lachesis, 2, 5 (F.) (two million five hundred thousand), by the bottom of the vial with her fingers' ends, at 4.2 P. M. Immediately prickling in the index finger. At 4.4 P. M., glimmering before the eyes. Pulse about 70, moderately full. It makes the chest wide. Deep breathing. It makes dizzy and agreeably cool. Sensation in the chest, as in a storm when on going against the wind one must breathe deeply. At 4.7 P. M., intermittence of the heart-stroke. Beating from the heart as far as the neck behind, above the shoulders. Pulse weak, almost imperceptible. Trembling of the heart. The medicine makes her feel light and easy. Whenever I feel her pulse she feels the pulse beating, but not otherwise. 4.11½ P. M., prickling in both feet, like asleep. Pulse very small and weak.

1868, July 22d. Mrs. S., 44 years old, the same who furnished the provings of Gelsemium 1 m (F.), published in the *North American Journal of Homœopathy*, February, 1867, page 413, and of Lachesis Cm (F.) presented to the American Institute of Homœopathy in 1867, however, though referred to the Committee on Publication, not printed in the *Transactions*, but in the *North American Journal of Homœopathy*, August, 1867, page 98.

At 2.54 P. M., she took a vial of Lachesis, 2, 3 (F.) in the hollow left hand, and said, this is indeed a warm glass. Coldness along the hand, disagreeable, gradually but weaker, ascending as far as the shoulder-joint. Fine sensation, as of a slit with ensuing burning at the lower thumb- and the wrist-joints. She remarks, it must be a very high potency, because everything goes on so easy and yet rapid. 3.2½ P. M., it always goes to the middle between the shoulder-blades, like a pressure, which then gently dissolves. Sometimes, like an agreeable coolness, it goes up the arm, where it is lost in the side, followed by a disagreeable heat, but the sensation is very fine. She feels only that it acts, but the action is so fine that it loses itself under the observation; feels it still some in the thumb.

Later on she took Lachesis 2, 2 (F.) in her left hand, and felt it immediately in the left arm. Burning in the left arm, especially in the forearm.

July 23d. A. M. In the night and by day the left arm became hot first, then the heat went into the mamma, a stitch into the very root of it, as on nursing when the nipple is sore and burning. This lasted all day, was worse in the evening, and went away in the night. At the same time the mamma felt stiff, and was hard to the touch.

July 23d. 3.19 P. M., took Lachesis 2, 2 (F.) in her left hand again. The vial throbs in the thumb, index and fourth fingers. Everything wants to go up the arm, but is stopped at the wrist. 3.26½ P. M., the vial becomes cooler, but it goes up the arm hot. In the pit of the stomach is something that hinders breaking, as if it were stopped up. Sighing. It passes up the left side of the neck, upon the left upper side of the occiput, where it feels like swollen and soft. 3.32 P. M., slight tremor through the whole body, with some anxiety, as if something touches the affections. From the pit of the stomach it went as far as the vertex, as if it would lift up the cranium, which did not succeed, and so it went down toward the nose, like an aura. The vial becomes warmer and where previously it went up hotter it now ascends cooler. The heat disappears and it is more agreeable. 3.38 P. M., puts the vial away, because she feels a spasmodic contraction of the third and fourth fingers of the right hand, starting from the first joints, at the same place where previously she had felt something similar in the left hand. Tiredness of the left arm, as if she had worked too hard, which lasted far into the night.

1868, July 12th. Miss C. F., 45 years old, took Lachesis 2 m (F.) in her left hand. After six minutes it makes her somewhat tired. Aching on the left top of the shoulder in the bone. Stiff in the neck. Momentary pain at a minute point at the left lower jaw near the angle. In the occiput near the neck stiffness with some pain, then going up with the sensation as if the head is drawn backwards. Sensation of heat going up the arm outside. Yawning. Sense of fatigue. She would like to lie down. Burning of the eyes. Occupancy (Eingenommenheit) of the head from the occiput upwards. Feet heavy and asleep from the knees down, as before a thunderstorm. Hands heavy. She feels as if greatly fatigued. Stiff on walking. Beaten-pain in the left inner upper arm, and in the two last right fingers. The spine between the shoulders feels like beaten as also the region in the left side of the thorax, between the clavicle and the middle breast-bone, going down at the left side of the chest. Yawning. On walking, sensation of heaviness in the legs, as if she could not move them as easily as usual. Aching in the neck and shoulder-blades, gradually going all over the upper part of the back. Feet feel like weights in them. Feverish, hot flush all over. So far it had lasted thirty minutes. She gradually recovered within another half-hour.

1868, July 30th. I had just finished Lachesis 2, 5 (F.) (two million five hundred thousand cent.), and Miss F. took the vial in her left hand at 8 P. M. The first symptom was sleepiness. Sensation on the vertex, as if it makes the head heavy, with some vertigo. Tearing drawing through the left fourth finger as far as the half fore-arm, as if she had *Lampyris splendidula* in the hand. Startling from the pit of the stomach. Earache on the left, then on the right side, pretty severe, the pain going down at the left side of the neck, then back before the ear toward the left temple. Rheumatic pain with stiffness in the right knee, on motion, after a few passes going out of the foot. Sleepiness. The heaviness on the vertex lasted several hours after the vial had been taken away.

In the night she woke up, and could not sleep. Becomes unusually hungry. Violent aching in both ears. Dull ache before both ears going down at the neck, with deafness, as if a skin were drawn over the ears.

August 1st. Slept well, but had a clairvoyant dream.

August 2d. Aching in the sacrum.

The foregoing observations are bare historical facts, which it is desirable to record in the literature of the homœopathic profession for future investigators who do not labor under the prejudices of the majority of the present time. They are facts obtained and preserved with the greatest care, and as such deserve to be considered with as much care by those who find in homœopathics the Science of Medicine.

